# Treatment appraisal and fate of HMs in up-flow anaerobic sludge blanket and trickling filter-based sewage treatment process: The case of a kaliti Centralized Wastewater Treatment Plant, Addis Ababa, Ethiopia

**DOI:** 10.1016/j.heliyon.2024.e34003

**Published:** 2024-07-04

**Authors:** Ashrake Hussen Shuralla, Andualem Mekonnen Hiruey, Getachew Dagnew Gebreeyessus

**Affiliations:** a Africa Center of Excellence for Water Management, College of Natural and Computational Science, Addis Ababa University, P.O. Box 1176, Addis Ababa, Ethiopia; b Center for Environmental Sciences, Addis Ababa University, P.O. Box 1176, Addis Ababa, Ethiopia; c Kotebe University of Education, P.O. Box 31248, Addis Ababa, Ethiopia

**Keywords:** Biological treatment system, Heavy metals, Physicochemical parameters, Sewage, Trickling filters, UASB

## Abstract

Heavy metals (HMs) in wastewater could pose a significant challenge to biological treatment systems such as in an Up-flow Anaerobic Sludge Blanket Reactor (UASBr) as well as Trickling Filter (TF) performances. These HMs are associated with retention and accumulation of solid precipitates, limitting solid-liquid separation, disrupting biochemical processes, which ultimately brings environmental risks, such as soil contamination and public health issues, dominantly due to the inhibited activities of degrading microorganisms. A cross-sectional study was applied to investigate the levels of HMs in sewage using composite and grab sampling taken from Kaliti Wastewater Treatment Plant and the samples were analyzed using Inductively Coupled Plasma Optical Emission Spectrometry (ICP-OES). The HMs concentrations in mean±(SD) were, Ag ranges from below detection level (BDL) to 63.5 (13.5) mg/kg; Ba 60 (4.47) μg/l to 1291(58.5) mg/kg; Al, BDL to 2358.5(662.5) mg/kg; Cd 0 μg/l to 0.35(0.15) mg/kg; Cr 0 μg/l to 10.5(0.7) mg/kg; Cu 0 μg/l to 23.9(1.2) mg/kg; Zn 5.45 (12.3) to 165(5.4) mg/kg, and Mn 165 (49.5) μg/l to 92.5(3.8) mg/kg. Results indicated that Kaliti Wastewater Treatment Plant was effective in removing pollutants and thereby meeting local and international discharge limits. The plant was also found to be effective in removing Al, Cd, Cu, and Cr, but not in removing Ba and Zn. However, a real time data collection and monitoring of seasonal physicochemical parameters and HM levels in the wastewater treatment plant is suggested useful.

## 1 Introduction

In fast urbanizing and expanding cities like Addis Ababa of Ethiopia [1]. wastewater treatment plants are essential aspects of the infrastructure. Effective wastewater treatment is vital for protecting public health and preserving environmental quality [2,3]. Technology selections for wastewater treatment can significantly influence the characteristics of the resulting sludge and effluent by-products [4]. With its limitations, Up-flow Anaerobic Sludge Blanket (UASB) in wastewater treatment plants (WWTPs) is among the popular anaerobic technologies favored mainly for its efficiency, low energy consumption as well as the lower sludge volume produced [5]. The case study, Kaliti Centralized Wastewater Treatment Plant (KWWTP) in Addis Ababa employs theis technology followed with trickiling filters and the final disinfection process to effectively handle the wastewater in part of the city [5].

Biological wastewater treatment aims to establish a procedure that makes it easier to collect and treat degradable organics in view of recover energy, water and soil nutrients [6,7]. However, HMs in wastewater or sewage pose a challenge for these biological systems because of their subsequent accumulation and toxicity nature [8]. Consequently, the effluent and sludge discharged from such WWTPs cause harmful environmental effects [9], with effluent polluting water and damaging biodiversity, and sludge containing contaminants like heavy metals that lead to soil toxicity and necessitate proper disposal [[10], [11], [12]]. Typical origins of heavy metals (HMs) in urban wastewater consist of industrial emissions, runoff from storm water, and the household sewage itself [13]. The treatment and fate of these contaminants in wastewater treatment processes are of great concern with time and urbanization [14]. Though UASB reactors are renowned for their capacity to efficiently handle highly concentrated organic wastewater [15], trickling filters (TF) offer a sturdy secondary treatment that lowers organic loads and refines the wastewater even more [16], which are interests in this study.

Recent research has underscored both the challenges and opportunities related to the removal of HMs in wastewater treatment systems [13,14]. However, the removal of HMs in wastewater treatment systems is facing challenges like large-volume sludge formation, fouling and scaling, and concurrent ion removal [17]. In fact, HMs removal in such systems can improve water management, reduce handling costs [18] and promote sustainability by focusing on eco-friendly, cost-effective materials and methods [17]. Therefore, managing effluent and sludge as the by-products of the existing WWTPs, is imperative to mitigate environmental impact and ensure sustainable resource utilization [7]. Additionally, sludge management is increasingly becoming an environmental challenge because of the high energy costs associated with WWTP, accounting for a large portion of the operating costs. Therefore, it is essential to have an appropriate assessment methodology for suggesting possible sludge management options and thereby to determine whether pollution is being diverted from water into other media, like air or soil or not [19].

Kaliti Wastewater Treatment Plant (KWWTP) in Addis Ababa, Ethiopia, is a municipally owned WWTP treating the wastewater collected from the central part of the city. After the treatment operations the effluent is discharged into the Little Akaki River nearby, and the final sludge is pumped and gets dried using drying beds at site. Unfortunately, industrial wastes are also illegally released into the treatment plant system through sewer lines, generating various issues, despite the plant being designed to treat domestic waste [20]. as Also, the direct release of hospital and similar health institutions' effluents without proper pretreatment have presented substantial difficulties in the reciving Kaliti treatment plant [21]. These issues could lead mostly to the presence of HMs in the effluent, which later brings a negative impact on agricultural soil, when used for irrigation and other purposes. Despite the critical importance of understanding the treatment performance of such WWTPs, there is a significant gap in knowledge, particularly concerning HMs. Addressing this gap is perceived essential for gaining a comprehensive insight into the plant's efficacy and the associated possible public and environmental health risks.

Therefore, this study focus mainly to evaluate the effectiveness of the Up-flow Anaerobic Sludge Blanket reactor (UASBr) and the TF-based sewage treatment processes in Addis Ababa, with a particular focus on the removal and fate of HMs. Theis specifically focuses on assessing the performances of the UASB and TF systems in reducing organic pollutants and HMs from incoming wastewater and also investigating the accumulation and distribution of HMs in the sludge produced by these treatment systems. Furthermore, recommendations for optimizing the treatment processes to enhance the removal of HMs and mitigate their environmental effects are proposed. Indeed, results obtained will significantly contribute to ongoing environmental safeguard practices by emphasizing the importance of adhering to environmental regulations to controlling pollution from domestic waste. Thus, it assists in protecting the public and environmental health and providing data-driven insights towards enhancing environmental compliance as well as in realizing sustainable practices. Additionally, it offers useful perspectives and methods that can be utilized in comparable areas dealing with the issue of HMs pollution in wastewater.

## 2 Materials and methods

### 2.1 Study area description

**Fig. 1:**
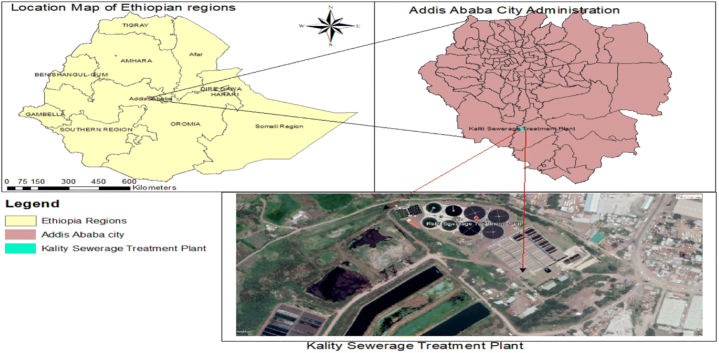
Locating Kaliti Centralized Wastewater Treatment Plant (KWWTP).

Addis Ababa, the capital and largest city of Ethiopia, serves as the political, economic, and cultural hub of the country and Africa. The initial centralized wastewater collection system in Addis Ababa was implemented during the 1960s with the purpose of gathering and transporting wastewater to a treatment plant situated near Kaliti, which was then considered the southern boundary of the city. KWWTP was built in the 1970s and expanded in 2018 with funding from the World Bank. It is located in Addis Ababa’s Kaliti neighborhood on the east side of the little Akaki River with coordinate’s 8°54′52″N38°45′18″E. The KWWTP was constructed with the intention of treating 100,000 cubic meters of sewage each day. However, it currently operates at about 70 % of that design capacity, treating roughly 70,000 cubic meters per day, a reduction primarily due to the incomplete connectivity of the city's sewage infrastructure to the plant. Wastewater from approximately 1800 connections into the 120 km large sewer network and from other sources is transported to the wastewater treatment plant every day. The sewer system was designed for 200,000 households but only serves about 13,000 households (Fig. 1).

**Fig. 2:**
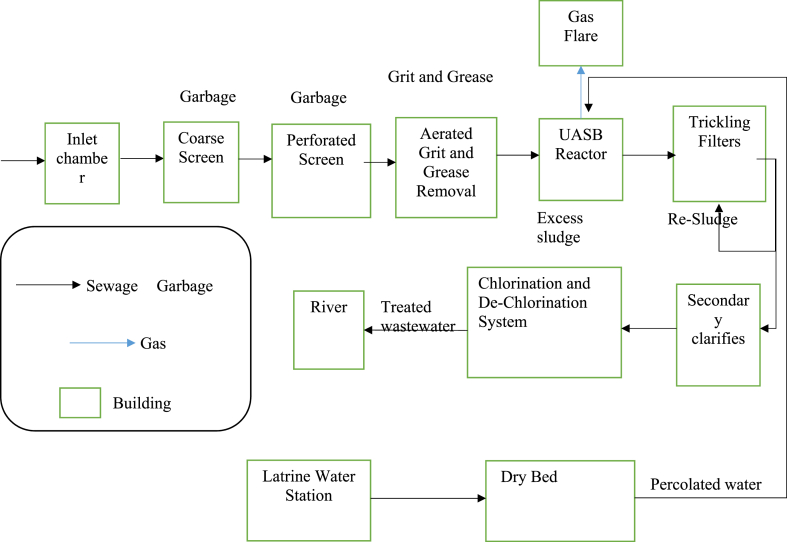
KWWTP process flow diagram.

KWWTP consists of a preliminary treatment system, biological treatments (UASB & TF), and a disinfection process (Fig. 2). The primary treatment stage helps separate suspended solids, grease, and heavy inorganic solids, reducing BOD_5_ by 15–30 %. The UASB treatment stage consists of 20 UASB reactor cells in four lines of five cells each. The plant has a hydraulic retention time (HRT) of 12.2 h, volumetric hydraulic loading (VHL) of 1.97 m^3^/m^3^ d, organic loading rate (OLR) of 2.29 kg-COD/m^3^, and solid retention time (SRT) of 38 d. The treated effluent from the UASBr flows to the next treatment stage in the trickling filter distribution chambers.

The UASBr has estimated removal efficiencies for BOD_5_, COD, and TSS of 55 %, 55 %, and 70 %, respectively. The TFstage comprises four reinforced concrete filters with UV-resistant plastic media, drainage, and a ventilation system. The final sedimentation tank enters the center, where a circular well distributes the flow equally in all directions. The UASB reactors' stabilized excess sludge is directed to gravity beds, with a daily volume of 360 m^3^/d. The Treatment Plant's sludge drying process takes one to two weeks, reaching a maximum concentration of 35 %, indicating reduced water content and increased solid material. It has 26 beds, with 30 cm UASB and 40 cm latrine sludge layers.

### 2.2 Sampling

To investigate the presence and concentrations of HMs and other more physicochemical parameters in wastewater, the current study adopted a cross-sectional design with repetitive measurements. The research was conducted during two distinct seasons: April and May (dry) and June (wet), allowing for the examination of seasonal variations. Samples were meticulously collected from raw wastewater, treated effluents, and sludge across different units of the wastewater treatment system. This comprehensive approach ensured a thorough representation of the system's performance and allowed for the identification of potential sources of contamination. During a 24-h period, composite samples of influent and effluent were gathered continuously, ensuring a comprehensive understanding of the system's dynamics. Additionally, grab samples were taken from critical points within the treatment process, including UASBr, TFs, secondary clarifiers, and drying beds.

To maintain sample integrity and prevent contamination, this study employed acid-washed polyethylene bottles for sample collection. This choice was made based on their inert nature and ability to preserve sample quality during transport and storage. Upon collection, physicochemical parameters were promptly analyzed on the same day to ensure data accuracy and reliability. Conversely, samples designated for HM analysis were transported to the laboratory on the same day and were stored at 4 °C until analysis. This approach minimizes the risk of sample degradation and preserved the integrity of the HM constituents.

### 2.3 Analytics

The on-site measurement of physicochemical parameters, such as pH and conductivity, was conducted using portable meters using Hana portable pH meter and conductivity meters respectively. Various tests were performed, including COD chromium-sulphoric acid oxidation in Hach Lange method, the five days’ biological oxygen demand (BOD_5_) Manometric Hach Method for Measuring BOD_5_, conductivity Method APHA 2510 B, TSS APHA 2540 D method, ammonium-nitrogen (NH^+^_4_-N) Indo-phenol blue Hach Lange method, and total phosphate (TP) Phosphor Molybdenum Blue Hach Lange method. These parameters are important in directing suitable treatment and management techniques by offering useful information on the several aspects of water quality, such as organic and inorganic pollutants, nutrient levels, conductivity, and suspended solids content. Samples were collected and analyzed for total HM concentration using ICP-OES (5000X), with concentrations ranging from parts per billion (ppb) to parts per million (ppm). It is suitable for liquid samples, solid samples after digestion, and gaseous samples (via nebulization). A microwave digester (MARS 6240/50) was used to process the sludge samples according to EPA Method 3051A before analysis.

### 2.4 Data analysis

IBM SPSS Statistics version 25 was used to conduct mean comparisons, variation, and correlation analyses among physicochemical parameters and HMs. Following the analysis, Tukey’s post hoc test was utilized for multiple comparisons to identify significant differences between groups. Descriptive results were subsequently generated using Microsoft excel and are visually presented in tables and figures to facilitate interpretation and visualization of the findings. A variety of quality assurance measures were used to ensure quality assurance in the study, including standardized, calibrated, and triplicate testing methods. To assess the validity of the analysis for physicochemical parameters and HMs, data quality assurance, assumption testing, reproducibility of statistical analysis, and result interpretation were used. A sensitivity analysis was conducted using variable study periods, parameter variations, and outlier sensitivity.

## 3 Results and discussions

### 3.1 Physicochemical characteristics of wastewater treated at the KWWTP

**Fig. 3:**
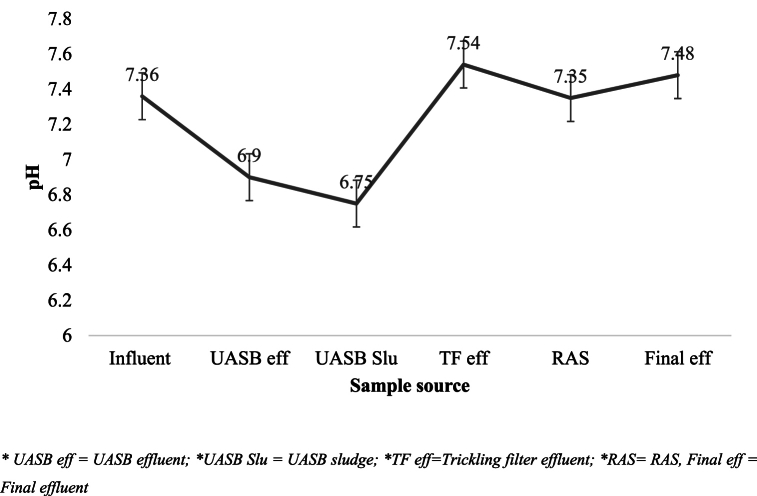
Mean values of pH along sewage treatment units.

The measured pH values in wastewater samples taken from various treatment units ranged from 6.75 ± 0.04 to 7.54 ± 0.13 in UASBr sludge and TF effluent respectively (Fig. 3). The mean pH for the UASB sludge was 6.75, while that for the TF effluent was 7.54, which varied significantly (P < 0.005). A similar study conducted by Maurya and Srivastava [22] to assess seasonal variation on physicochemical characteristics in the Jaganpur WWTP in India in summer (mid-March to mid-July) found out that the pH of effluent samples varied significantly along the different sampling sites. It ranged from 8.5 to 9, which is alkaline in nature. The pH of the treated final effluent water also varied from 7.45 to 8.33 in a WWTP in Bhopal (India) [23]. Hammoudani et al. [24] reported that inlet (raw wastewater) pH ranges from 7.89 ± 0.078 while effluent ranges from 7.35 ± 0.05.

The slightly alkaline pH of the TF effluent can be attributed to the nitrification process, in which nitrifying bacteria convert ammonia to nitrite and nitrate, resulting in an increase in pH. This alkaline shift is a crucial component in wastewater treatment operations. The pH of the WWTP UASB sludge was found to be 6.75, indicating a mildly acidic nature. This can result from the generation of organic acids by the anaerobic digestion process [25]. However, this slight acidity does not necessarily hinder the process, as anaerobic bacteria are capable of not only surviving but also thriving under mildly acidic conditions. However, it was discovered that the pH of the TF effluent was 7.54, which is fairly alkaline. This could be due to the bicarbonates that are created during aerobic digestion [25]. This was in agreement with the pH value (5.85–7.55) observed in WWTPs in Cape Town, South Africa. The pH value of the final treated effluent ranged from 6 to 9, with a mean pH below the discharge limit of the EEPA [26]. Adherence to the specified pH limit in effluent is crucial as it directly impacts treated effluent, treatment plant, and receiving water quality, causing scaling, pipe corrosion, and aquatic life damage [25].

**Fig. 4:**
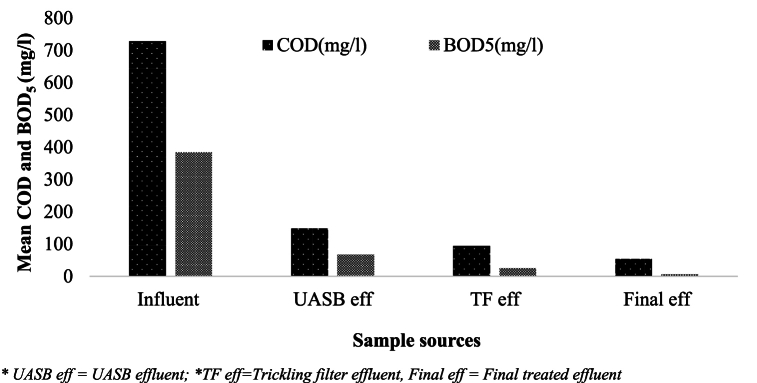
Mean values of BOD_5_ and COD (mg/l) along the WWTP units.

The mean concentration of BOD_5_ in the influent was 383.7 ± 74.58 mg/l, ranging from 210 to 740 mg/l. Meanwhile, BOD_5_ in the final effluent had a mean concentration of 6 ± 0.73 mg/l and was in the range of 4–8 mg/l, which is consistent with the values from the literature (Fig. 4).

The highest BOD5 concentration (383.7 ± 74.58 mg/l) was recorded as expected in the influent, whereas the lowest concentration (6 ± 0.73 mg/l) was noted in the final effluent and significantly varied (P < 0.005). The WWTP results align with previous studies on BOD5 variations during the sample period, such as one conducted in India on home wastewater treatment [27]. The WWTP was efficient in eliminating organic debris that decomposes biodegradable and thereby enhancing effluent quality. The BOD_5_ level in the final effluent was within the EEPA recommended effluent discharge limit into the surface water body (60 mg/l) [26].

The COD concentrations in the wastewater received by the treatment plant have a mean value of 728.3 ± 121.03 mg/l, which matches the value from the literature (Table 1). Mean COD concentrations showed significant variations (P < 0.005), with the smallest being 53 ± 2.21 mg/l in final treated effluent and the largest being (728.3 ± 121.03 mg/l) in influent. A study by Bhave [27] has found that untreated wastewater had COD levels between 188 and 684.6 mg/l, whereas treated wastewater had COD levels of 15–112.8 mg/l. The influent wastewater’s COD concentration can range from 200 to 500 mg/l, while the effluent wastewater’s concentration can be as low as 20–30 mg/l [28]. The mean COD concentration of the treated effluent was less than 250 mg/l, which is below the EEPA discharge limit for water bodies [26].

**Table 1:** Features of wastewater treatment in many cities across the world.

Parameters	Study location					
	Pakistan [29]		Palestine Al Bireh [30]	Brazil Pedrega [31]	Colombia Cali [31]	Netherland Bennekom [31]
	Karachi	Lahore				
BOD	220–475	200–215		368	95	231
COD tot	200–1400	580–803	1586	727	267	520
Sulfate	50–200	–	–	18	–	15
NH_4_^+^-N	–	–	80	34	17	–
TN	–	–	104	44	24	–
TP	–	–	13	11	13	18
TSS	250–900	106–176	736	492	215	–

**Fig. 5:**
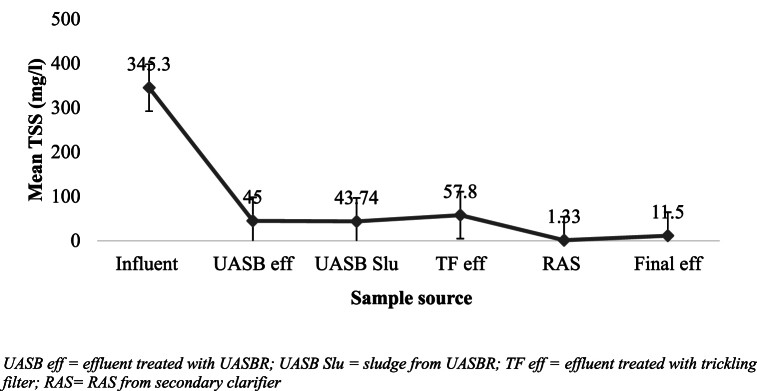
Mean values of TSS in the WWTP across the treatment unit.

The mean TSS concentration was 345.3 ± 61.9 mg/l and 11.5 ± 1.1 mg/l in the influent and treated effluent, respectively, which was significantly varied at P < 0.005. This result was found to be consistent with that of Bhave [27], reporting TSS concentrations in treated wastewater to vary from 10 to 48 mg/l across different test sites, whereas untreated wastewater can have concentrations ranging from 38 to 500 mg/l. The maximum TSS concentration in the KWWTP was obviously recorded in the influent (345.33 ± 61.9 mg/l), whereas the minimum was recorded in the RAS (1.33 ± 0.19 mg/l) (Fig. 5) and was found to be significant at P < 0.005. The minimal concentration in the RAS can be attributed to extended, similar rainfall conditions throughout study period that have a substantial effect on the TSS concentration in the KWWTP. The effluent of the WWTP had a mean TSS concentration of 11.5 ± 1.1 mg/l, which was below the provisional EEPA discharge limits into water bodies [26].

**Fig. 6:**
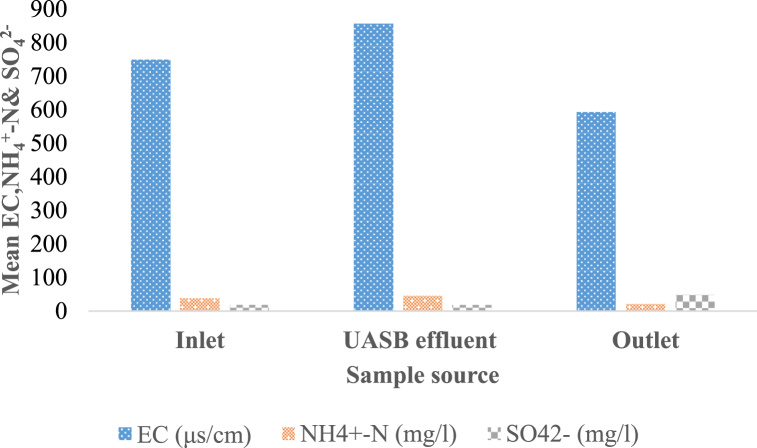
Average values of EC, NH_4_^+^-N, and SO_4_^2−^ across wastewater treatment units.

The measured EC value of the wastewater in the treated and UASB effluents of the treatment plant was between 591.8 ± 31.21 and 855.3 ± 43.7 μs/cm (Fig. 6), respectively, and was consistent with the literature (Table 1). The lower EC value in the treated effluent compared with the UASB effluent was due to the fact that the TF unit reduces the dissolved salts and minerals by biological oxidation and nitrification. However, the lower EC value also means that the treated effluent has a lower buffering capacity and may be more susceptible to pH changes. The measured mean concentration of EC was significantly varied across sampling units at P < 0.005. It could be caused by variations in the type and amount of inorganic materials, such as sulfides, carbonate compounds, chlorides, and dissolved solids, during various stages of the treatment unit (Bressani-Ribeiro et al., 2018). The mean wastewater treatment effluent concentration (591.83 ± 31.21 mg/l) was below the EEPA's preliminary discharge limits for aquatic bodies [26].

In a related study conducted by Maurya and Srivastava [22] to evaluate the seasonal variation physicochemical characteristics in the Jaganpur WWTP in India, the EC of effluent samples fluctuated from 200 to 250 μS/cm across different sampling sites during the summer (mid-March to mid-July), with the lowest levels recorded in the winter (mid-November to mid-March). During the rainy periods, higher EC levels are likely caused by increased volume of water consumption, leading to higher domestic wastewater discharge, higher flow rates from water bodies, and lower flow rates of wastewater treatment effluent. Conversely, reduced EC concentrations in winter occur due to the low overflow runoff from the WWTP and improved treatment of ions.

The treatment plant daily receives waste containing SO_4_^2−^ at concentrations between 11.3 and 26.1 mg/l, with an average concentration of 17.98 ± 2.54 mg/l (Fig. 6), aligning with values in Table 1 from the literature. The UASB effluent had a minimum sulfate concentration of 17.9 mg/l and a maximum of 48.4 mg/l. This difference was statistically significant at P < 0.005. The increase in the sulfate concentration in the UASBr and TF based WWTP is mainly due to the oxidation of sulfide produced by the anaerobic degradation of organic matter in the UASB reactor. The sulfide is stripped from the UASB effluent and reacts with oxygen in the air or in the TF, forming sulfate. The concentration of sulfate in the effluent can increase due to several reasons, such as the use of sulfate-containing chemicals in industrial processes or the presence of sulfate in the environment [32]. The WWTP's final effluent contained 48.4 ± 2.65 mg/l of sulfate, which was discharged into the little Akaki River, below the EEPA's provisional discharge limit [26].

The final treated effluent and UASB effluent at the KWWTP had average daily concentrations of NH_4_^+^–N ranging from 20.9 ± 2.56 mg/l to 45.42 ± 4.14 mg/l, respectively (Fig. 6). This finding aligns with Nelson et al. [33], who found that the ammonium nitrogen concentration in wastewater ranges from 20 to 40 mg/l, which indicates a greater fecal matter content, which is produced through metabolic processes in plants, animals, and humans. The UASB effluent recorded the highest mean concentration of ammonium nitrogen (45.4 ± 4.14 mg/l), whereas the final treated effluent recorded the lowest (20.9 ± 2.56 mg/l). The main reason for the high concentration of NH^+^_4_ –N in UASBr is that it transforms wastewater into a more soluble form of nitrogen, requiring post-treatment to reduce NH_4_^+^ –N concentration and prevent eutrophication and toxicity issues [34]. However, the mean treated effluent concentration of ammonium nitrogen (20.9 ± 2.56 mg/l) was below the acceptable discharge limit into water bodies [26]. The total nitrogen (TN) concentration in a WWTP significantly varied P < 0.005 from 58.17 ± 3.79 mg/l in the influent to 52.17 ± 4.24 mg/l in the treated final effluent. This value is lower than the literature value in Palestine Al Bireh [30] but higher than the values in Brazil and Colombia [31] Table 1. The TN concentration in the WWTP influent decreases as nitrogen is removed through biological processes such as nitrification and denitrification. The final treated effluent of the WWTP had a mean concentration of TN 52.17 ± 4.24 mg/l, which is below the provisional discharge standard limit of 80 mg/l set by EEPA for water bodies [20]. However, regular monitoring is still necessary to prevent eutrophication in the receiving water bodies.

The Total phosphorus (TP) value of the WWTP ranged from 26.35 ± 1.77 mg/l to 27.7 ± 2.31 mg/l at the influent and treated effluent, respectively. KWWTP, despite lower literature values in Palestine [29], Brazil, and Colombia [31], lacks significant phosphorus removal, necessitating alternative techniques like membrane filtering, chemical precipitation, or biological phosphorus removal. If not, the receiving water bodies may be at risk of eutrophication and deterioration of water quality due to the high TP value of the effluent.

The mean TP concentration in the treated effluent increased from 26.35 ± 1.77 mg/l in influent to 27.7 ± 2.31 mg/l, which is below the national EEPA 10 (mg/l) recommended value [26]. The release of phosphorus from the anaerobic sludge in the UASBr is the primary cause of the increase in TP content in the effluent [35]. The bacteria in the sludge retain extra phosphorus when exposed to aerobic conditions and release it when exposed to anaerobic ones and this behavior is known as luxury uptake [36].

### 3.2 Removal efficiency of the KWWTP for physicochemical pollutants

**Fig. 7:**
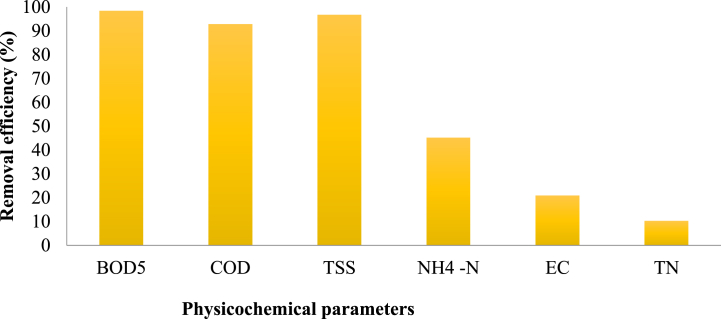
Removal efficiency based on the physicochemical parameters by the WWTP.

Based on the data shown in (Fig. 7), the KWWTP exhibited varying levels of removal efficiency, achieving high efficiency for BOD5, COD, and TSS, while displaying lower efficiency for EC and TN. The findings indicate that a WWTP system utilizing UASB followed by a trickling filter is a viable option for treating domestic sewage. Chernicharo, C.A.L. et al. [37] also reported similar findings on the viability of a pilot-scale UASB/trickling filter-based WWTP system for domestic sewage treatment. This information can be beneficial for researchers and engineers involved in implementing similar systems, offering insights into the operational conditions, performance, and potential challenges of UASB and trickling filter-based treatment processes.

The Bahirdar textile WWTP’s BOD_5_ removal efficiency was approximately 92.07 %, surpassing the EEPA’s recommended criteria [38]. By the current study, the COD removal efficiency of the treatment plant ranged from 86.74 % to 95.2 %, with a mean removal efficiency of 92.72 % which leads to significant decrease COD concentration (Fig. 7). In contrast, a research carried out by Abebaw [39] at KWWTP revealed that the treatment plant's mean COD removal effectiveness was roughly 73 %, which is within the EEPA's permitted discharge limits into water bodies. The Bahirdar Dar Textile WWTP had a COD removal efficiency of approximately 62.27 %, which is below the guidelines set by the EEPA to discharge into water bodies [20,30]. Over the course of the investigation, the BOD_5_ removal efficiency of the KWWTP ranged from 97.5 % to 99.46 %, with no discernible variations (Fig. 7).

Additionally, the treatment plant’s TSS removal effectiveness ranged from 92.38 % to 98.22 %, with a mean of 96.67 % (Fig. 7). During the dry season, the KWWTP showed a TSS reduction efficiency of 64 %, which is good [39]. A similar study conducted by Bhave [27] on domestic sewage treatment plants found the maximum and minimum removal efficiencies of TSS at 97.62 % and 89.47 %, respectively. The Bahirdar textile WWTP achieved an impressive TSS reduction efficiency of 71.96 % [38]. KWWTP had an ammonium removal efficiency of 45.2 % as of ammonium nitrogen, which was below the designed performance. The effectiveness of removing ammoniacal nitrogen from wastewater is determined by the treatment process and the initial concentration of ammoniacal nitrogen [40]. Study conducted by varga et al. [41] on (UASB) and Constructed Wetland (CW) system treating municipal wastewater found high removal efficiencies for certain metals, with the order of removal being Sn > Cr > Cu > Pb > Zn > Fe, achieving removal rates between 63 and 94 % and Medium removal efficiencies were observed for Ni (49 %), Hg (42 %), and Ag (40 %), while Mn and As showed negative percentage removals.

### 3.3 Concentration of HMs across treatment unit in the studied treatment plant

**Fig. 8:**
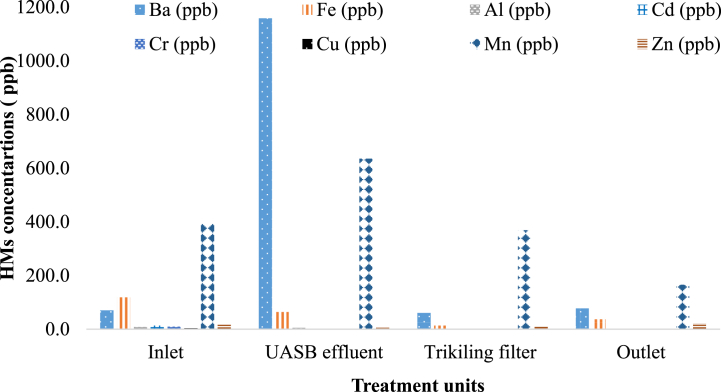
Mean concentration of heavy metals in different treatment units of KWWTP.

The laboratory analysis of the wastewater from treatment plant demonstrated 100 % detection for Zn, Cr, Cd, Fe, Ba, and Cu and 83.33 % detection rate for Al. None of the samples contained Pb or Ag. The influent samples had an average concentration of HMs ranging from BDL for Ag and Pb to 395 ± 13.10 μg/l for Mn (Fig. 8). Several studies have reported similar results, with no Pb found in the influents of WWTP [42]. The very low Pb level (<0.001) suggests a reduction in industrial discharge over the study period. This could be due to decreased contribution from human-made sources to the treatment plant or a dilution effect from increased rainfall. The highest average concentration of HMs found in the KWWTP was Mn, with a concentration of 395 ± 13.10 μg/l, whereas the lowest concentration was Cu, with a concentration of 3.33 ± 2.1 μg/l and significantly varied at P < 0.005. The increased Mn concentration can be attributed to domestic activities and natural sources. According to Adeyinka’s research [43], Fe had the highest concentration of all HMs, with a mean value ranging from 0.122 to 1.808 mg/l in the influent samples.

The average concentration of HMs in the UASB effluent ranged from BDL for Ag and Pb to 1158.33 ± 1072.34 μg/l for Ba. The amounts of HMs in the KWWTP effluent from the TF varied between BDL for Ag and Pb to 368.33 ± 85.18 μg/l for Mn over the sampling periods (P < 0.05). The higher concentration of Mn in the TF effluent may be due to operational factors such as pH and oxidation– reduction, as well as a higher concentration of Mn in the WWTP influent. The dominant metal in effluent was Mn, while Cr, Cu, and Cd had the lowest concentrations. The higher concentration of Mn in the treated effluent may be due to the influence of Mn concentration in the influent of the KWWTP and operational parameters such as pH, which can affect Mn dissolution. The homogeneity of variance test (P = 0.00) showed that the amount of HMs in the treated effluent did not vary significantly, indicating that the treatment plant is highly efficient. However, it should be noted that the treated effluent still contained higher amounts of Ba (60 μg/l), Fe (36.667 μg/l), Mn (165 μg/l), and Zn (22 μg/l) after treatment. These metals can accumulate in soil irrigated by this water and can be transferred to plants and animals through the food chain. On average, the concentrations of HMs in the treated effluent were significantly lower than the levels set by the EEPA for surface water discharge (Table 2).

**Table 2:** Mean ± standard error values of HMs in sludge samples and comparison with international standard values for agricultural application.

HMs	Influent (μg/l)	UASB sludge in mg/kg	Returned sludge in mg/kg	Dry sludge in mg/kg	USEPA (mg/kg)	Final effluent (μg/l)	EEPA standard in mg/kg
Ag	BDL	BDL	BDL	1.27 ± 0.27		BDL	
Ba	70 ± 2.58	0.07 ± 0.0028	0.0767 ± 0.0099	25.82 ± 0.83		60 ± 4.47	
Fe	118.33 ± 22.718	275.202 ± 119.264	0.067 ± 0.049	2167.88 ± 435.27		36.67 ± 26.79	
Al	8 ± 2	5.042 ± 2.224	0.03	47.067 ± 13.25		BDL	
Cd	13.33 ± 4.22	0.0067 ± 0.0049	0.00016	0.0067 ± 0.0024	85	0	0.5
Cr	9.33 ± 3.7	0.0267 ± 0.0096	0	0.021 ± 0.012	3000	0	500
Cu	3.33 ± 2.1	0.06 ± 0.0242	0	0.48 ± 0.016	4300	0	500
Mn	395 ± 13.1	0.382 ± 0.105	1.567 ± 0.225	1.85 ± 0.053		165 ± 49.5	
Zn	20 ± 12.38	0.42 ± 0.149	0.0267 ± 0.0067	3.3 ± 0.108	7500	22 ± 7.85	500
Pb	BDL	BDL	BDL	BDL		BDL	

The mean HMs concentration in UASB sludge of KWWTP was dominated by Fe (275.2 ± 119.26 mg/l) and Cd (0.0067 ± 0.0049 mg/l), which took the lowest concentration. The concentration of Fe in UASB sludge might be due to corrosion of pipes and addition of Fe due to biological processes such as the breakdown of organic matter by Fe-reducing bacteria, whereas the lowest concentration of Cd might be due to the nature of Cd in the natural system and low industrial input that leads to exist at the lowest concentration. The sludge can precipitate or adsorb Fe from the wastewater, lowering the Fe concentration in the effluent [44]. The high concentration of Fe in the UASB sludge may indicate that Fe is being effectively removed from the wastewater during the treatment process, whereas the lowest concentration of Cd in the UASB sludge is a positive outcome, indicating successful removal or minimal input of Cd in the wastewater treatment process. Sludge with a high Fe concentration may be more difficult to dewater, reuse, or dispose of because of its increased volume and metal content [44].

The Mn concentration (1.567 ± 0.225 mg/l) was dominant in the RAS, whereas Cr and Cu had the lowest mean concentrations in the sludge. Mn is a HM that can have adverse effects on the microbial ecology of activated sludge [45]. A related study suggests that HMs such as Mn can impact the biological nutrient removal process by affecting the microbial community’s metabolic activity [45]. The same study also highlights the importance of monitoring HM concentrations in influent wastewater to support decision-making at the wastewater plant level [45].

The occurrence and distribution of HMs in dry sludge accounts 90 % of the HM distribution. This indicates that HMs are getting accumulated in the sludge. Fe (16538.5 ± 15,192 mg/kg) dominated in the dry sludge, whereas Cd (0.35 ± 0.15 mg/kg) exhibited the lowest concentration Table 2. The higher concentration of Fe in the dry sludge might be due to the higher concentration of Fe in UASBr, which results from different sources in the city. The findings were in line with those of Duan et al. [46], who found that the mean concentration of Cd in sewage sludge from municipal WWTPs in Shanxi, China, which is the lowest.

The study revealed that the HMs in dry sludge from WWTP was below the standards of United States environmental protection agency (USEPA), World health organization (WHO) and EEPA required for agricultural application Table 2. In KWWTP, HMs, which are absent in the treated effluent, tend to accumulate in the sludge, whereas metals that are low concentration but high effluents tend to attract liquids. HMs play a crucial role in anaerobic digestion, precipitating as sulfides, carbonates, and hydroxides, and sorbing to the solid fraction, including biomass or inert particulate matter. The concentrations of Ba, Fe, Zn, Mn, Cd, Cr, Cu, and Ag are relatively low in the liquid part of the sewage but reasonably high in the sludge. This indicates that these HMs precipitate under the prevailing conditions in sewage and concentrate in the sludge. The KWWTP sludge was found to have a relatively greater content of HMs. Addressing the higher contaminant concentrations in the sludge requires a comprehensive approach involving influent characterization, process optimization, proper sludge management, and robust monitoring and regulation. Based on the available research possible solutions for heavy metal laden sludge to use some beneficial application includes Stabilization and Solidification (S/S) [47], Phytoremediation [48], Chemical extraction [49], Thermal treatment [50] and Landfilling with Proper Liner Systems [51]. A comprehensive risk assessment should be conducted to ensure that the chosen solution effectively treats the sludge and minimizes potential risks to human health and the environment.

### 3.4 Heavy metal removal efficiency

**Fig. 9:**
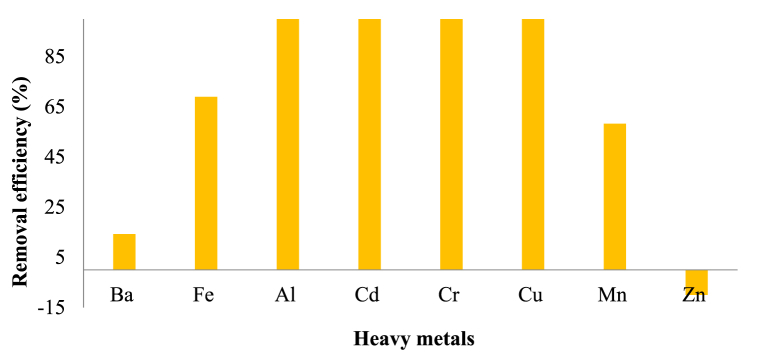
Comparison of heavy metals removal efficiencies in KWWTP.

As depicted in Fig. 9, KWWTP demonstrated high removal efficiencies for aluminum (Al), cadmium (Cd), chromium (Cr), and copper (Cu), moderate removal efficiency for iron (Fe) and manganese (Mn), and lower removal efficiency for zinc (Zn) and barium (Ba). This assessment is important in comprehending how well the UASB and TF-based treatment process can remove HMs, a vital step in guaranteeing the treated wastewater's safety and safeguarding environmental health. The treatment plant achieved 100 % removal efficiency for Al, Cd, Cr, and Cu, effectively eliminating these metals despite not being specifically engineered for metal removal (Fig. 9). Considering the effluent concentration and removal efficiency for Mn and Fe, the relatively high discharge of may result from the high initial concentration, whereas the relatively high discharge of Ba and Zn may result from the poor efficiency of the treatment (Fig. 9). However, the removal efficiency for Ba was quite low, at 14.29 %, indicating that the treatment process was not effective in removing this metal from the wastewater. The results for Cu (88.88 %) and Cr (91.98 %) found in this study were higher than the results reported by Feng et al. [52]. Surprisingly, the KWWTP exhibited lowest removal efficiencies when it came to removing Zn, indicating its ineffectiveness in addressing this particular contaminant. The treatment plant also removed Fe and Mn from the wastewater with removal efficiencies of 69.01 % and 58.23 %, respectively.

During the study period, it was observed that the removal efficiency of the WWTP was ineffective, as it removed less than 50 % of the total influx of Ba (14.28). It is worth noting that Zhou et al. [53] reported negative removal efficiency for Zn, Pb, Cd, and Ni in a China WWTP. Meanwhile, Xiao et al. [54] found that a full-scale hybrid constructed wetland receiving municipal sewage had average removal efficiencies of 46.5 %, −7.3 %, −25.6 %, and 13.8 % for Cu, Pb, Cr, and Zn, respectively.

In this study, it was found that NH^+^_4_-N had a positive correlation with BOD_5_ (0.924**) and COD (0.817*), whereas EC had a positive correlation with BOD_5_ (0.968**), NH_4_^+^ –N (0.962**), and COD (0.812*). This correlation indicates that organic pollution might contribute to NH_4_^+^ –N pollution in wastewater. Monitoring and controlling the levels of NH^+^_4_ –N in the wastewater can reduce organic pollution and improve water quality. The findings reveal a noteworthy and affirmative connection between TSS and COD (0.964**) during wastewater treatment, indicating a strong association between them (Table 3). However, there is no indication of any link between TSS and the concentration of HMs, indicating that other factors may be responsible for the presence of HMs in wastewater. The presence of organic matter and suspended solids are typical factors that impact both TSS and COD, which explains the robust correlation between them. This is different from Koju et al. [55] who found a positive relationship between TSS and the concentration of HMs, specifically Fe, Cu, Mn, Zn, Pb, and As.

**Table 3:** Correlation analysis of physicochemical parameters and HMs in KWWTP.

	pH	BOD5	COD	NH_4_–N	TSS	EC	SO^2-^_4_	TP	TN	Inflow	Ba	Fe	Al	Cd	Mn	Zn	
																	Cr
pH	1																
BOD	−0.75	1															
COD	−0.157	0.73	1														
NH^+^_4 -_N	−0.576	.924^a^	.817^b^	1		.											
TSS	−0.144	0.678	.964^a^	0.708	1												
EC	−0.691	.968^a^	.812^b^	.962^a^	0.746	1											
SO_4_^2−^	0.513	−0.661	−0.599	−0.556	−0.559	−0.721	1										
TP	0.066	0.478	0.51	0.54	0.363	0.397	−0.112	1									
TN	−0.324	0.466	0.05	0.277	0.031	0.242	0.059	0.664	1								
Inflow	−0.659	0.409	−0.232	0.173	−0.184	0.205	0.137	0.119	0.781	1							
Ba	0.014	−0.19	−0.455	−0.331	−0.323	−0.374	0.722	−0.036	0.545	0.697	1						
Fe	−0.045	−0.255	−0.171	−0.05	−0.277	−0.057	−0.146	−0.516	−0.801	−0.514	−0.625	1					
Al	0.404	−0.705	−0.41	−0.517	−0.423	−0.525	0.202	−0.686	−0.914^b^	−0.671	−0.378	.838^b^	1				
Cd	−0.408	−0.027	−0.212	−0.258	−0.026	−0.01	−0.32	−0.817^b^	−0.341	0.131	0	0.186	0.231	1			
Mn	−0.546	0.549	0.12	0.247	0.242	0.35	−0.123	0.218	0.789	.838^b^	0.591	−0.79	−0.857^b^	0.241	1		
Zn	0.302	0.127	0.632	0.169	0.638	0.256	−0.639	0.13	−0.396	−0.733	−0.732	0.078	0.111	0.06	−0.288	1	
Cr	−0.173	0.613	0.682	0.708	0.488	0.663	−0.672	0.712	0.177	−0.239	−0.696	0.061	−0.315	−0.483	−0.123	0.516	1

a Correlation is significant at the 0.01 level (2-tailed).

b Correlation is significant at the 0.05 level (2-tailed).

Al showed a strong negative correlation with TN (−0.914*) and a strong positive correlation with Fe (0.838*) (Table 3). Cd showed a significantly negative correlation with TP (−0.914*). The observation in treatment plants revealed a significant negative correlation between Al and TN. This correlation is attributed to the hindrance caused by Al ions in the availability of nitrogen compounds, thereby impeding the assimilation process for microorganisms and the formation of complex compounds. Furthermore, the strong positive correlation between Al and Fe can be attributed to their mutual presence in industrial processes and co-precipitation in similar forms. It is also worth noting that certain ligands could increase their adsorption onto solid particles. The Mn exhibited a strong negative correlation with Al (−0.857*) and a positive correlation with inflow (0.838*) (Table 3). This strong negative correlation between Mn and Al may be attributed to competition for adsorption sites on the surfaces of solids within the treatment plant and chemical reactions. A study conducted by Pipi et al. [56] in Brazil found that high concentrations of metals in the aquatic environment can significantly affect the pH, chemical oxygen demand, and dissolved oxygen of the treated water [57]. The study also found that the abundance of HMs such as Ba, Mn, Zn, Cu, Se, Fe, and Al in the samples was correlated with the physicochemical parameters of the treated water [57].

## 4 Conclusions

In summary, the physicochemical characteristics of KWWTP effluent were generally below the EEPA discharge limit. The WWTP successfully reduced most of the physicochemical parameters, including BOD_5_, COD, TSS, NH_4_^+^-N, TN, and EC. The concentrations of HMs in the sewage effluent and sludge were below the discharge limits set by EEPA and WHO and also met USEPA standards for land application and surface disposal. Among the HMs analyzed, Ba, Fe, Zn, Mn, Cd, Cr, Cu, and Ag had low concentrations in the liquid sewage but higher concentrations in the sludge, indicating precipitation and concentration of HMs in the sludge. The WWTP was highly effective in removing Al, Cd, Cu, and Cr, but less effective in removing Ba and Zn. The study also found positive correlations between BOD_5_ and COD, COD and TSS, and HM concentrations in the treatment plant. Negative correlations were observed between TN and Al, TP and Cd, and Al and Mn. However, the study suggests the regular analysis of HMs in treatment plants during the dry season, and implementing regular monitoring to prevent environmental pollution. In addition, the study recommends assessing As and Hg levels in WWTP, researching emerging contaminants, and exploring new approaches for sludge management and resource recovery.

## Funding

Not applicable.

## Data availability statement

This study's data has not been placed in a repository that is open to the public. The information in this article and the supporting materials can be reviewed and examined further. We recognize that research data sharing is critical to enabling assessment, expanding on findings, and boosting confidence in scholarly publications.

## CRediT authorship contribution statement

**Ashrake Hussen Shuralla:** Writing – original draft, Investigation. **Andualem Mekonnen Hiruey:** Validation, Supervision, Conceptualization. **Getachew Dagnew Gebreeyessus:** Writing – review & editing, Supervision, Methodology.

## Declaration of competing interest

The authors declare that they have no known competing financial interests or personal relationships that could have appeared to influence the work reported in this paper.
